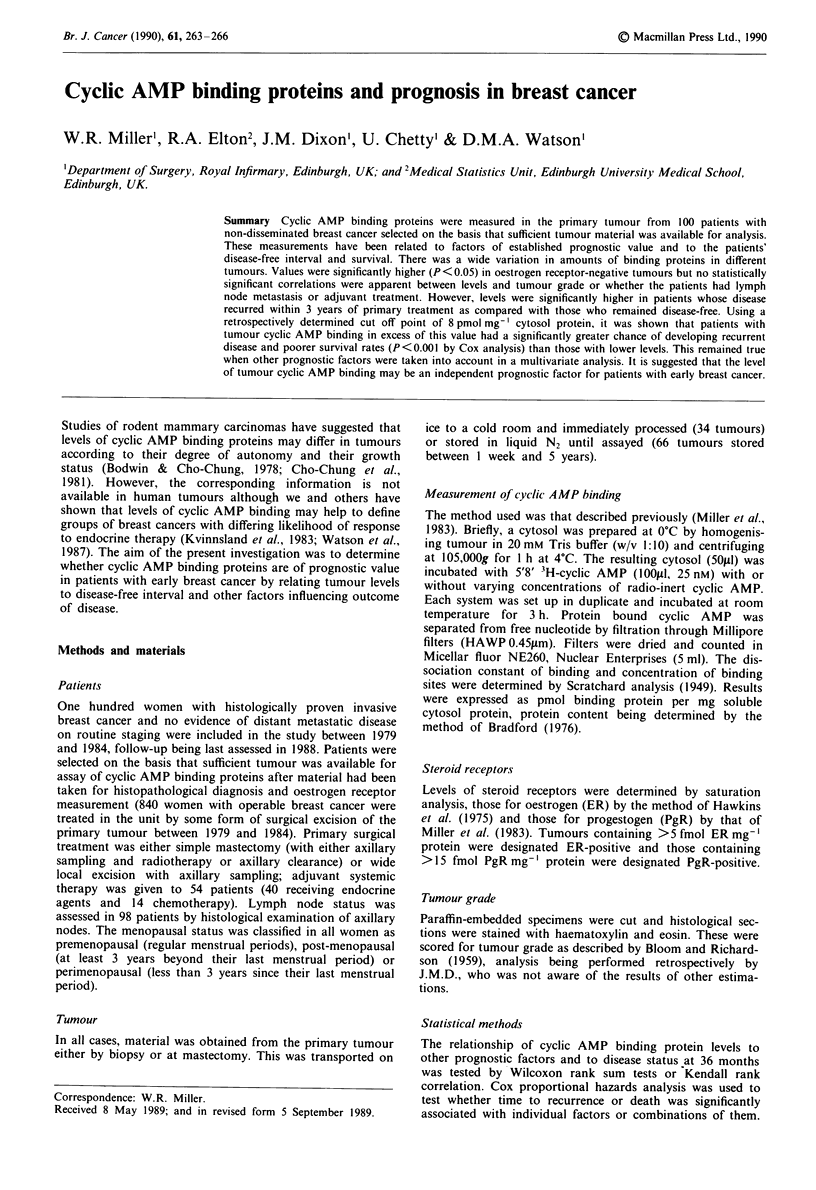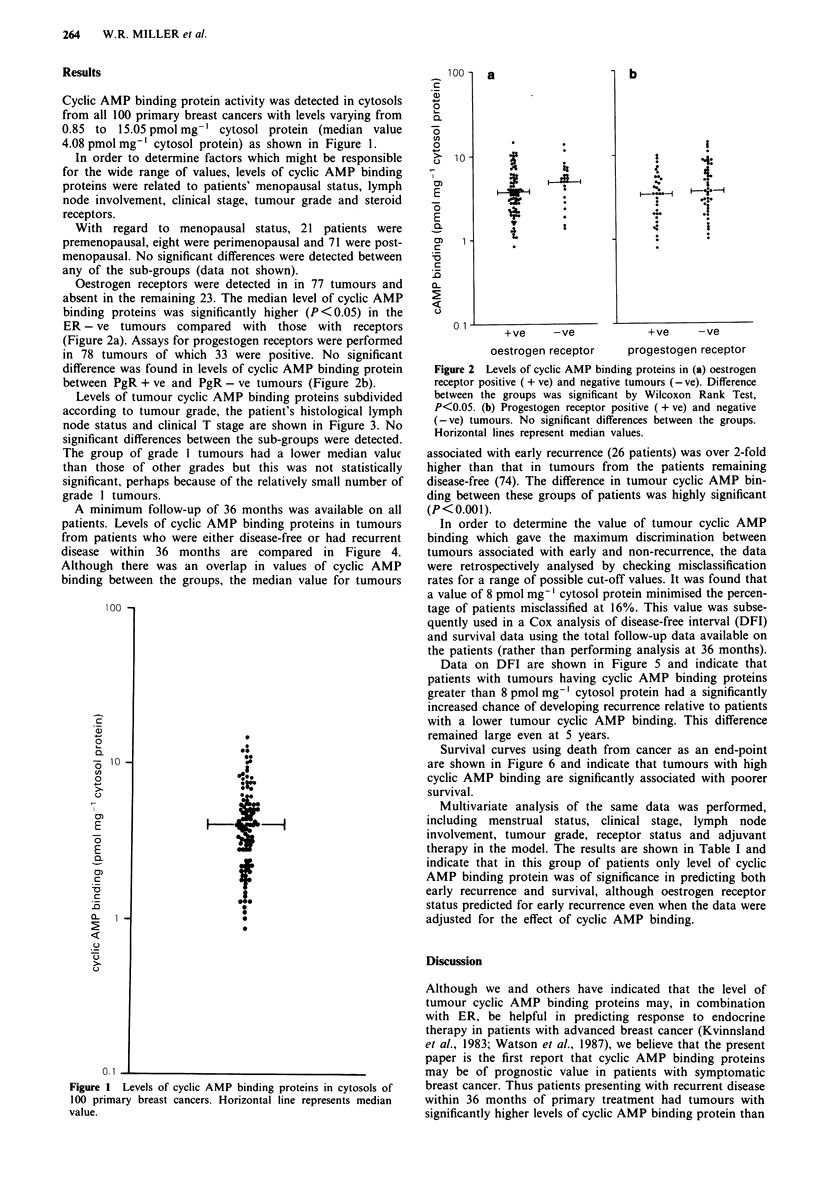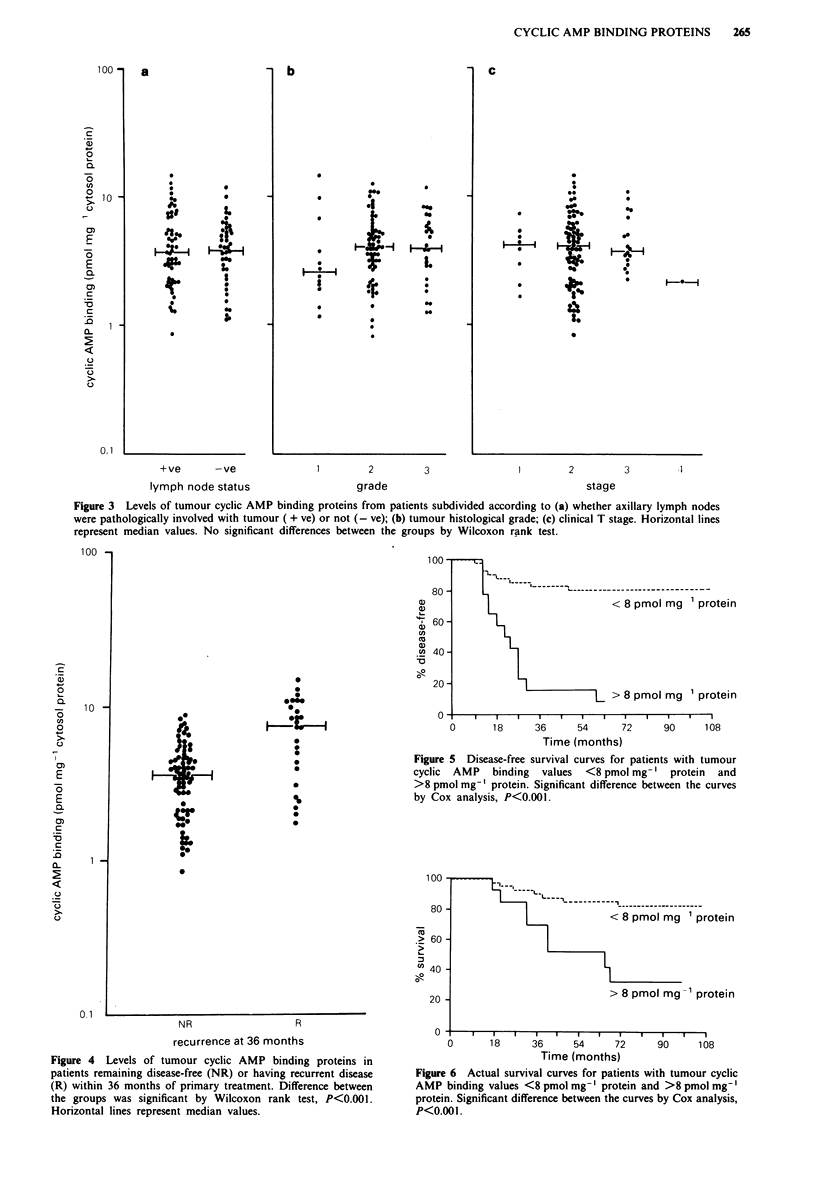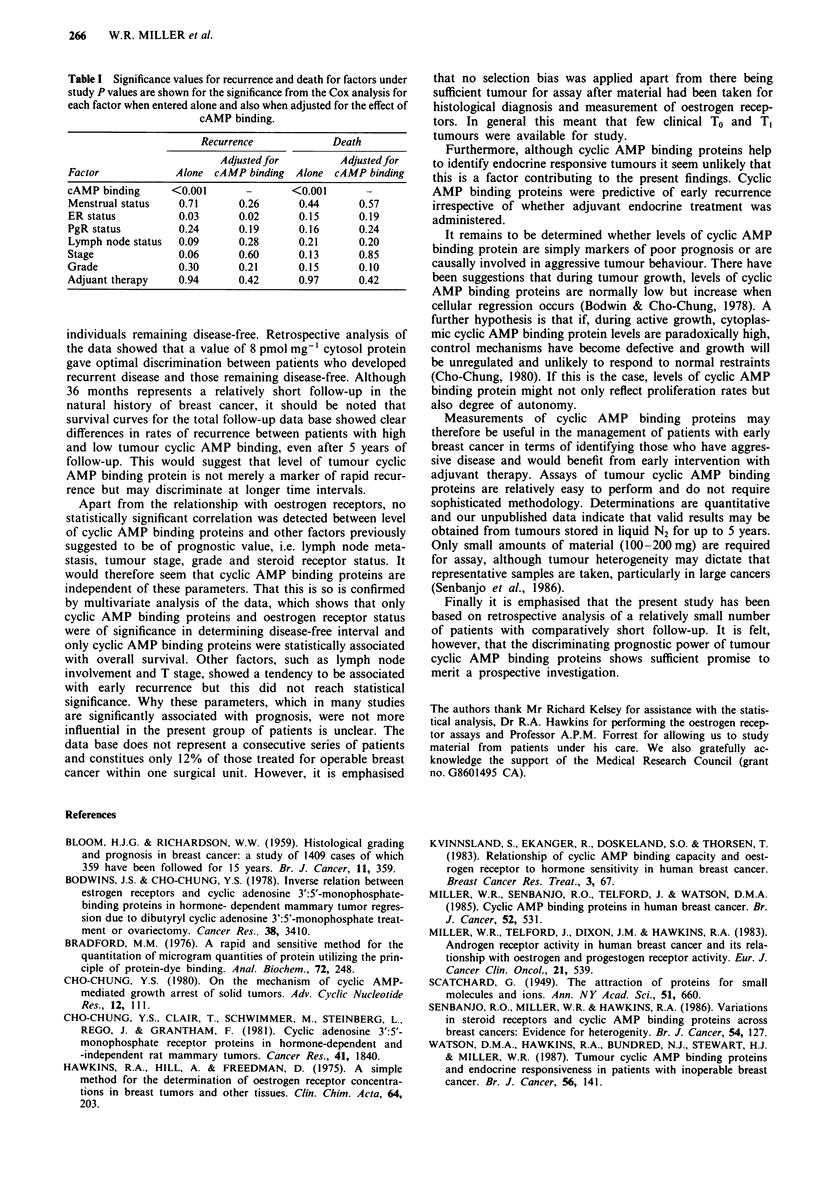# Cyclic AMP binding proteins and prognosis in breast cancer.

**DOI:** 10.1038/bjc.1990.48

**Published:** 1990-02

**Authors:** W. R. Miller, R. A. Elton, J. M. Dixon, U. Chetty, D. M. Watson

**Affiliations:** Department of Surgery, Royal Infirmary, Edinburgh, UK.

## Abstract

Cyclic AMP binding proteins were measured in the primary tumour from 100 patients with non-disseminated breast cancer selected on the basis that sufficient tumour material was available for analysis. These measurements have been related to factors of established prognostic value and to the patients' disease-free interval and survival. There was a wide variation in amounts of binding proteins in different tumours. Values were significantly higher (P less than 0.05) in oestrogen receptor-negative tumours but no statistically significant correlations were apparent between levels and tumour grade or whether the patients had lymph node metastasis or adjuvant treatment. However, levels were significantly higher in patients whose disease recurred within 3 years of primary treatment as compared with those who remained disease-free. Using a retrospectively determined cut off point of 8 pmol mg-1 cytosol protein, it was shown that patients with tumour cyclic AMP binding in excess of this value had a significantly greater chance of developing recurrent disease and poorer survival rates (P less than 0.001 by Cox analysis) than those with lower levels. This remained true when other prognostic factors were taken into account in a multivariate analysis. It is suggested that the level of tumour cyclic AMP binding may be an independent prognostic factor for patients with early breast cancer.


Br. J. Cancer (1990), 61, 263-266 © Macmillan Press Ltd., 1990

### Cyclic AMP binding proteins and prognosis in breast cancer

W.R. Miller[1], R.A. Elton[2], J.M. Dixon[1], U. Chetty[1] & D.M.A. Watson[1]

[1]*Department of Surgery, Royal Infirmary, Edinburgh, UK; and* [2]*Medical Statistics Unit, Edinburgh University Medical School, Edinburgh, UK.*

**Summary** Cyclic AMP binding proteins were measured in the primary tumour from 100 patients with non-disseminated breast cancer selected on the basis that sufficient tumour material was available for analysis. These measurements have been related to factors of established prognostic value and to the patients' disease-free interval and survival. There was a wide variation in amounts of binding proteins in different tumours. Values were significantly higher ($P < 0.05$) in oestrogen receptor-negative tumours but no statistically significant correlations were apparent between levels and tumour grade or whether the patients had lymph node metastasis or adjuvant treatment. However, levels were significantly higher in patients whose disease recurred within 3 years of primary treatment as compared with those who remained disease-free. Using a retrospectively determined cut off point of 8 pmol mg$^{-1}$ cytosol protein, it was shown that patients with tumour cyclic AMP binding in excess of this value had a significantly greater chance of developing recurrent disease and poorer survival rates ($P < 0.001$ by Cox analysis) than those with lower levels. This remained true when other prognostic factors were taken into account in a multivariate analysis. It is suggested that the level of tumour cyclic AMP binding may be an independent prognostic factor for patients with early breast cancer.

Studies of rodent mammary carcinomas have suggested that levels of cyclic AMP binding proteins may differ in tumours according to their degree of autonomy and their growth status (Bodwin & Cho-Chung, 1978; Cho-Chung *et al.*, 1981). However, the corresponding information is not available in human tumours although we and others have shown that levels of cyclic AMP binding may help to define groups of breast cancers with differing likelihood of response to endocrine therapy (Kvinnsland *et al.*, 1983; Watson *et al.*, 1987). The aim of the present investigation was to determine whether cyclic AMP binding proteins are of prognostic value in patients with early breast cancer by relating tumour levels to disease-free interval and other factors influencing outcome of disease.

### Methods and materials

### *Patients*

One hundred women with histologically proven invasive breast cancer and no evidence of distant metastatic disease on routine staging were included in the study between 1979 and 1984, follow-up being last assessed in 1988. Patients were selected on the basis that sufficient tumour was available for assay of cyclic AMP binding proteins after material had been taken for histopathological diagnosis and oestrogen receptor measurement (840 women with operable breast cancer were treated in the unit by some form of surgical excision of the primary tumour between 1979 and 1984). Primary surgical treatment was either simple mastectomy (with either axillary sampling and radiotherapy or axillary clearance) or wide local excision with axillary sampling; adjuvant systemic therapy was given to 54 patients (40 receiving endocrine agents and 14 chemotherapy). Lymph node status was assessed in 98 patients by histological examination of axillary nodes. The menopausal status was classified in all women as premenopausal (regular menstrual periods), post-menopausal (at least 3 years beyond their last menstrual period) or perimenopausal (less than 3 years since their last menstrual period).

### *Tumour*

In all cases, material was obtained from the primary tumour either by biopsy or at mastectomy. This was transported on ice to a cold room and immediately processed (34 tumours) or stored in liquid $N_2$ until assayed (66 tumours stored between 1 week and 5 years).

### *Measurement of cyclic AMP binding*

The method used was that described previously (Miller *et al.*, 1983). Briefly, a cytosol was prepared at 0°C by homogenising tumour in 20 mM Tris buffer (w/v 1:10) and centrifuging at 105,000*g* for 1 h at 4°C. The resulting cytosol (50µl) was incubated with 5'8' $^3$H-cyclic AMP (100µl, 25 nM) with or without varying concentrations of radio-inert cyclic AMP. Each system was set up in duplicate and incubated at room temperature for 3 h. Protein bound cyclic AMP was separated from free nucleotide by filtration through Millipore filters (HAWP 0.45µm). Filters were dried and counted in Micellar fluor NE260, Nuclear Enterprises (5 ml). The dissociation constant of binding and concentration of binding sites were determined by Scratchard analysis (1949). Results were expressed as pmol binding protein per mg soluble cytosol protein, protein content being determined by the method of Bradford (1976).

### *Steroid receptors*

Levels of steroid receptors were determined by saturation analysis, those for oestrogen (ER) by the method of Hawkins *et al.* (1975) and those for progestogen (PgR) by that of Miller *et al.* (1983). Tumours containing $>5$ fmol ER mg$^{-1}$ protein were designated ER-positive and those containing $>15$ fmol PgR mg$^{-1}$ protein were designated PgR-positive.

### *Tumour grade*

Paraffin-embedded specimens were cut and histological sections were stained with haematoxylin and eosin. These were scored for tumour grade as described by Bloom and Richardson (1959), analysis being performed retrospectively by J.M.D., who was not aware of the results of other estimations.

### *Statistical methods*

The relationship of cyclic AMP binding protein levels to other prognostic factors and to disease status at 36 months was tested by Wilcoxon rank sum tests or Kendall rank correlation. Cox proportional hazards analysis was used to test whether time to recurrence or death was significantly associated with individual factors or combinations of them.

Correspondence: W.R. Miller.
Received 8 May 1989; and in revised form 5 September 1989.

### Results

Cyclic AMP binding protein activity was detected in cytosols from all 100 primary breast cancers with levels varying from 0.85 to 15.05 pmol $mg^{-1}$ cytosol protein (median value 4.08 pmol $mg^{-1}$ cytosol protein) as shown in Figure 1.

In order to determine factors which might be responsible for the wide range of values, levels of cyclic AMP binding proteins were related to patients' menopausal status, lymph node involvement, clinical stage, tumour grade and steroid receptors.

With regard to menopausal status, 21 patients were premenopausal, eight were perimenopausal and 71 were postmenopausal. No significant differences were detected between any of the sub-groups (data not shown).

Oestrogen receptors were detected in in 77 tumours and absent in the remaining 23. The median level of cyclic AMP binding proteins was significantly higher ($P<0.05$) in the ER − ve tumours compared with those with receptors (Figure 2a). Assays for progestogen receptors were performed in 78 tumours of which 33 were positive. No significant difference was found in levels of cyclic AMP binding protein between PgR + ve and PgR − ve tumours (Figure 2b).

Levels of tumour cyclic AMP binding proteins subdivided according to tumour grade, the patient's histological lymph node status and clinical T stage are shown in Figure 3. No significant differences between the sub-groups were detected. The group of grade 1 tumours had a lower median value than those of other grades but this was not statistically significant, perhaps because of the relatively small number of grade 1 tumours.

A minimum follow-up of 36 months was available on all patients. Levels of cyclic AMP binding proteins in tumours from patients who were either disease-free or had recurrent disease within 36 months are compared in Figure 4. Although there was an overlap in values of cyclic AMP binding between the groups, the median value for tumours associated with early recurrence (26 patients) was over 2-fold higher than that in tumours from the patients remaining disease-free (74). The difference in tumour cyclic AMP binding between these groups of patients was highly significant ($P<0.001$).

**Figure 1** Levels of cyclic AMP binding proteins in cytosols of 100 primary breast cancers. Horizontal line represents median value.

**Figure 2** Levels of cyclic AMP binding proteins in (**a**) oestrogen receptor positive ( + ve) and negative tumours ( − ve). Difference between the groups was significant by Wilcoxon Rank Test, $P<0.05$. (**b**) Progestogen receptor positive ( + ve) and negative ( − ve) tumours. No significant differences between the groups. Horizontal lines represent median values.

In order to determine the value of tumour cyclic AMP binding which gave the maximum discrimination between tumours associated with early and non-recurrence, the data were retrospectively analysed by checking misclassification rates for a range of possible cut-off values. It was found that a value of 8 pmol $mg^{-1}$ cytosol protein minimised the percentage of patients misclassified at 16%. This value was subsequently used in a Cox analysis of disease-free interval (DFI) and survival data using the total follow-up data available on the patients (rather than performing analysis at 36 months).

Data on DFI are shown in Figure 5 and indicate that patients with tumours having cyclic AMP binding proteins greater than 8 pmol $mg^{-1}$ cytosol protein had a significantly increased chance of developing recurrence relative to patients with a lower tumour cyclic AMP binding. This difference remained large even at 5 years.

Survival curves using death from cancer as an end-point are shown in Figure 6 and indicate that tumours with high cyclic AMP binding are significantly associated with poorer survival.

Multivariate analysis of the same data was performed, including menstrual status, clinical stage, lymph node involvement, tumour grade, receptor status and adjuvant therapy in the model. The results are shown in Table I and indicate that in this group of patients only level of cyclic AMP binding protein was of significance in predicting both early recurrence and survival, although oestrogen receptor status predicted for early recurrence even when the data were adjusted for the effect of cyclic AMP binding.

### Discussion

Although we and others have indicated that the level of tumour cyclic AMP binding proteins may, in combination with ER, be helpful in predicting response to endocrine therapy in patients with advanced breast cancer (Kvinnsland et al., 1983; Watson et al., 1987), we believe that the present paper is the first report that cyclic AMP binding proteins may be of prognostic value in patients with symptomatic breast cancer. Thus patients presenting with recurrent disease within 36 months of primary treatment had tumours with significantly higher levels of cyclic AMP binding protein than

Figure 3 Levels of tumour cyclic AMP binding proteins from patients subdivided according to (a) whether axillary lymph nodes were pathologically involved with tumour ( + ve) or not (− ve); (b) tumour histological grade; (c) clinical T stage. Horizontal lines represent median values. No significant differences between the groups by Wilcoxon rank test.

Figure 4 Levels of tumour cyclic AMP binding proteins in patients remaining disease-free (NR) or having recurrent disease (R) within 36 months of primary treatment. Difference between the groups was significant by Wilcoxon rank test, $P<0.001$. Horizontal lines represent median values.

Figure 5 Disease-free survival curves for patients with tumour cyclic AMP binding values $<8$ pmol mg$^{-1}$ protein and $>8$ pmol mg$^{-1}$ protein. Significant difference between the curves by Cox analysis, $P<0.001$.

Figure 6 Actual survival curves for patients with tumour cyclic AMP binding values $<8$ pmol mg$^{-1}$ protein and $>8$ pmol mg$^{-1}$ protein. Significant difference between the curves by Cox analysis, $P<0.001$.

**Table I** Significance values for recurrence and death for factors under study *P* values are shown for the significance from the Cox analysis for each factor when entered alone and also when adjusted for the effect of cAMP binding.

| | *Recurrence* | | *Death* | |
|---|---|---|---|---|
| *Factor* | *Alone* | *Adjusted for cAMP binding* | *Alone* | *Adjusted for cAMP binding* |
| cAMP binding | <0.001 | - | <0.001 | - |
| Menstrual status | 0.71 | 0.26 | 0.44 | 0.57 |
| ER status | 0.03 | 0.02 | 0.15 | 0.19 |
| PgR status | 0.24 | 0.19 | 0.16 | 0.24 |
| Lymph node status | 0.09 | 0.28 | 0.21 | 0.20 |
| Stage | 0.06 | 0.60 | 0.13 | 0.85 |
| Grade | 0.30 | 0.21 | 0.15 | 0.10 |
| Adjuant therapy | 0.94 | 0.42 | 0.97 | 0.42 |

individuals remaining disease-free. Retrospective analysis of the data showed that a value of 8 pmol $mg^{-1}$ cytosol protein gave optimal discrimination between patients who developed recurrent disease and those remaining disease-free. Although 36 months represents a relatively short follow-up in the natural history of breast cancer, it should be noted that survival curves for the total follow-up data base showed clear differences in rates of recurrence between patients with high and low tumour cyclic AMP binding, even after 5 years of follow-up. This would suggest that level of tumour cyclic AMP binding protein is not merely a marker of rapid recurrence but may discriminate at longer time intervals.

Apart from the relationship with oestrogen receptors, no statistically significant correlation was detected between level of cyclic AMP binding proteins and other factors previously suggested to be of prognostic value, i.e. lymph node metastasis, tumour stage, grade and steroid receptor status. It would therefore seem that cyclic AMP binding proteins are independent of these parameters. That this is so is confirmed by multivariate analysis of the data, which shows that only cyclic AMP binding proteins and oestrogen receptor status were of significance in determining disease-free interval and only cyclic AMP binding proteins were statistically associated with overall survival. Other factors, such as lymph node involvement and T stage, showed a tendency to be associated with early recurrence but this did not reach statistical significance. Why these parameters, which in many studies are significantly associated with prognosis, were not more influential in the present group of patients is unclear. The data base does not represent a consecutive series of patients and constitues only 12% of those treated for operable breast cancer within one surgical unit. However, it is emphasised that no selection bias was applied apart from there being sufficient tumour for assay after material had been taken for histological diagnosis and measurement of oestrogen receptors. In general this meant that few clinical $T_0$ and $T_1$ tumours were available for study.

Furthermore, although cyclic AMP binding proteins help to identify endocrine responsive tumours it seem unlikely that this is a factor contributing to the present findings. Cyclic AMP binding proteins were predictive of early recurrence irrespective of whether adjuvant endocrine treatment was administered.

It remains to be determined whether levels of cyclic AMP binding protein are simply markers of poor prognosis or are causally involved in aggressive tumour behaviour. There have been suggestions that during tumour growth, levels of cyclic AMP binding proteins are normally low but increase when cellular regression occurs (Bodwin & Cho-Chung, 1978). A further hypothesis is that if, during active growth, cytoplasmic cyclic AMP binding protein levels are paradoxically high, control mechanisms have become defective and growth will be unregulated and unlikely to respond to normal restraints (Cho-Chung, 1980). If this is the case, levels of cyclic AMP binding protein might not only reflect proliferation rates but also degree of autonomy.

Measurements of cyclic AMP binding proteins may therefore be useful in the management of patients with early breast cancer in terms of identifying those who have aggressive disease and would benefit from early intervention with adjuvant therapy. Assays of tumour cyclic AMP binding proteins are relatively easy to perform and do not require sophisticated methodology. Determinations are quantitative and our unpublished data indicate that valid results may be obtained from tumours stored in liquid $N_2$ for up to 5 years. Only small amounts of material (100–200 mg) are required for assay, although tumour heterogeneity may dictate that representative samples are taken, particularly in large cancers (Senbanjo *et al.*, 1986).

Finally it is emphasised that the present study has been based on retrospective analysis of a relatively small number of patients with comparatively short follow-up. It is felt, however, that the discriminating prognostic power of tumour cyclic AMP binding proteins shows sufficient promise to merit a prospective investigation.

The authors thank Mr Richard Kelsey for assistance with the statistical analysis, Dr R.A. Hawkins for performing the oestrogen receptor assays and Professor A.P.M. Forrest for allowing us to study material from patients under his care. We also gratefully acknowledge the support of the Medical Research Council (grant no. G8601495 CA).

### References

BLOOM, H.J.G. & RICHARDSON, W.W. (1959). Histological grading and prognosis in breast cancer: a study of 1409 cases of which 359 have been followed for 15 years. *Br. J. Cancer*, **11**, 359.

BODWINS, J.S. & CHO-CHUNG, Y.S. (1978). Inverse relation between estrogen receptors and cyclic adenosine 3':5'-monophosphate-binding proteins in hormone- dependent mammary tumor regression due to dibutyryl cyclic adenosine 3':5'-monophosphate treatment or ovariectomy. *Cancer Res.*, **38**, 3410.

BRADFORD, M.M. (1976). A rapid and sensitive method for the quantitation of microgram quantities of protein utilizing the principle of protein-dye binding. *Anal. Biochem.*, **72**, 248.

CHO-CHUNG, Y.S. (1980). On the mechanism of cyclic AMP-mediated growth arrest of solid tumors. *Adv. Cyclic Nucleotide Res.*, **12**, 111.

CHO-CHUNG, Y.S., CLAIR, T., SCHWIMMER, M., STEINBERG, L., REGO, J. & GRANTHAM, F. (1981). Cyclic adenosine 3':5'-monophosphate receptor proteins in hormone-dependent and -independent rat mammary tumors. *Cancer Res.*, **41**, 1840.

HAWKINS, R.A., HILL, A. & FREEDMAN, D. (1975). A simple method for the determination of oestrogen receptor concentrations in breast tumors and other tissues. *Clin. Chim. Acta*, **64**, 203.

KVINNSLAND, S., EKANGER, R., DOSKELAND, S.O. & THORSEN, T. (1983). Relationship of cyclic AMP binding capacity and oestrogen receptor to hormone sensitivity in human breast cancer. *Breast Cancer Res. Treat.*, **3**, 67.

MILLER, W.R., SENBANJO, R.O., TELFORD, J. & WATSON, D.M.A. (1985). Cyclic AMP binding proteins in human breast cancer. *Br. J. Cancer*, **52**, 531.

MILLER, W.R., TELFORD, J., DIXON, J.M. & HAWKINS, R.A. (1983). Androgen receptor activity in human breast cancer and its relationship with oestrogen and progestogen receptor activity. *Eur. J. Cancer Clin. Oncol.*, **21**, 539.

SCATCHARD, G. (1949). The attraction of proteins for small molecules and ions. *Ann. NY Acad. Sci.*, **51**, 660.

SENBANJO, R.O., MILLER, W.R. & HAWKINS, R.A. (1986). Variations in steroid receptors and cyclic AMP binding proteins across breast cancers: Evidence for heterogenity. *Br. J. Cancer*, **54**, 127.

WATSON, D.M.A., HAWKINS, R.A., BUNDRED, N.J., STEWART, H.J. & MILLER, W.R. (1987). Tumour cyclic AMP binding proteins and endocrine responsiveness in patients with inoperable breast cancer. *Br. J. Cancer*, **56**, 141.